# Incidence, Predictors and Outcomes of Contrast Induced Nephropathy in Patients with ST Elevation Myocardial Infarction Undergoing Primary Percutaneous Coronary Intervention

**DOI:** 10.5334/gh.1071

**Published:** 2021-08-31

**Authors:** Mohamed Khalfallah, Amany Allaithy, Dina A. Maria

**Affiliations:** 1 Cardiovascular Department, Tanta University, EG

**Keywords:** Contrast induced nephropathy, ST elevation myocardial infarction, primary percutaneous coronary intervention

## Abstract

**Background:**

Contrast induced nephropathy (CIN) is considered one of the most common causes of hospital acquired renal failure and severely affects morbidity and mortality. Our objective was to investigate incidence, predictors and outcomes of CIN in patients with ST elevation myocardial infarction (STEMI) undergoing primary percutaneous coronary intervention (PPCI).

**Methods:**

The study was conducted on 550 patients with STEMI subjected to PPCI. Patients were classified into two groups according to the occurrence of CIN; group I (Patients without CIN) and group II (Patients with CIN). The two groups were assessed for the clinical outcomes including mortality and major adverse cardiac events (MACE).

**Results:**

Incidence of CIN was 10.6%, multivariate regression analysis identified the independent predictors of CIN including; age > 60 years OR 6.083 (CI95% 3.143–11.77, P = 0.001), presence of diabetes mellitus OR 2.491 (CI95% 1.327–4.675, P = 0.005), non-steroidal anti-inflammatory drugs (NSAIDs) use OR 2.708 (CI95% 1.393–5.263, P = 0.003), the volume of contrast agent >200 ml OR 6.543 (CI95% 3.382–12.65, P = 0.001) and cardiogenic shock OR 4.514 (CI95% 1.738–11.72, P = 0.002). Mortality was higher in group II than group I (11.9% vs. 4.4% respectively, P = 0.015). The incidence of MACE were higher in group II than group I (heart failure; 18.6% vs. 7.3%, cardiac arrest; 8.5% vs. 2.8% and cardiogenic shock; 16.9% vs. 6.9% with P. value = 0.003, 0.024, 0.007 respectively).

**Conclusion:**

Contrast induced nephropathy was associated with increased morbidity and mortality. The independent predictors of CIN were advanced age, diabetes mellitus, NSAIDs use, the volume of contrast agent >200 ml and cardiogenic shock.

## Introduction

The widespread adoption of primary percutaneous coronary intervention (PPCI) for the treatment of ST elevation myocardial infarction (STEMI), despite significantly improving cardiovascular outcomes, has increased the incidence of contrast induced nephropathy (CIN) due to the difficulties in assessing CIN risk and initiating prophylactic measures for prevention of CIN due to the emergency situation of STEMI, attendant hemodynamic compromise and the use of higher contrast volumes [1,2]. The administration of radiographic contrast media may lead to acute kidney injury (AKI) [3], which is called contrast induced acute kidney injury (CI-AKI) that is considered a reversible condition after exposure to contrast media. It occurs within 48 hours of contrast media exposure and recovery usually occurs within 7–10 days, with the majority of patients returning to their normal kidney function [4]. Pre-existent stage III chronic kidney disease (CKD), that is defined as an estimated glomerular filtration rate (e-GFR) <60 mL/min/1.73 m^2^ for greater than three months, is the most commonly recognized risk factor for CIN. However, CIN can occur in absence of underlying CKD if other risk factors are present [5].

Primary percutaneous coronary intervention is the preferred strategy of revascularization in patients with STEMI referred to hospitals with PCI capable facilities [6,7]. Contrast induced nephropathy can be preventable in those patients if the underlying risk factors for CIN are well identified and managed properly during the acute event [8,9]. Therefore, our objective was to determine the independent predictors for CIN development in patients with STEMI managed by PPCI. The wholly iatrogenic and predictable nature of CIN marks it as a cornerstone for ongoing cardiovascular and nephrology research, with a focus on pathophysiology, risk factors, and preventative, diagnostic and therapeutic measures.

## Patients and methods

The present study was conducted on 550 patients with STEMI who were managed by PPCI in our cardiovascular department. The patients were classified into two groups according to the occurrence of CIN; group I (Patients without CIN) and group II (Patients with CIN). The study was approved by the local research ethics committee of the faculty of medicine, Tanta University, and complied with the principles of the Declaration of Helsinki II. All patients who participated in the study signed a written informed consent and every patient had a code number pointed to his name, address and telephone number in a special file. ST elevation myocardial infarction was defined by the classic symptoms of typical chest pain, along with 1-mm ST-segment elevation in inferior leads, or 2-mm ST-segment elevation in the anterior chest leads occurring in two contiguous leads, or a new or presumably new left bundle branch block [10]. Patients with STEMI who received thrombolysis or underwent CABG or presented later after 24 hours were excluded from the study. We also excluded patients currently on renal dialysis at the time of their PCI.

All patients were subjected to detailed history taking about atherosclerosis risk factors e.g. hypertension, diabetes mellitus, smoking and dyslipidemia. History of comorbidities was inquired e.g. peripheral vascular disease (PVD), chronic kidney disease (CKD) (e-GFR ˂60 ml/min/1.73 m^2^) [11]. History of previous medication was interrogated including beta-blockers, angiotensin converting enzyme inhibitors/angiotensin receptor blockers, antiplatelet therapy, statins and non-steroidal anti-inflammatory drugs (NSAIDs) use that is defined as >1 dose/per week use of NSAIDs. Venous blood samples were taken from all patients pre-procedure and then 12, 24, 48 and 72 hours after the procedure, samples were used for measurement of serum creatinine and e-GFR based on Modification of Diet in Renal Disease (MDRD) equation in (ml/min/1.73 m^2^). Routine laboratory investigations including serum hemoglobin, random blood glucose, high-sensitivity cardiac troponin and CK-MB were measured to all patients. Patients were classified as normal kidney function (e-GFR ≥ 60 ml/min/1.73 m^2^), mild CKD (e-GFR = 45–59 ml/min/1.73 m^2^) moderate CKD (e-GFR = 30–44 ml/min/1.73 m^2^), or severe CKD (e-GFR ˂ 30 ml/min/1.73 m^2^) [11]. Transthoracic echocardiography was done to all patients.

On admission, patients received aspirin tablets 300 mg, clopidogrel 600 mg or ticagrelor 180 mg, in addition to intravenous unfractionated heparin. Primary PCI was done through trans-femoral or trans-radial artery approach. Standard left and right coronary angiograms were obtained for each patient. All patients were injected using a non-ionic low-osmolar contrast agent. Two experienced interventionists assessed the diagnostic coronary angiography. After identification of the anatomy, revascularization of the culprit lesion was done, the volume of contrast agent was measured and TIMI flow grade after the procedure was reported.

The primary endpoint of the study was the occurrence of CIN which is defined as a relative increase of serum creatinine level 25% or more or an absolute increase of 0.5mg/dl or more from baseline level at 48-72h following exposure to the contrast agent, in the absence of other explanation for the renal impairment [12]. The secondary endpoint was the occurrence of major adverse cardiac events (MACE) including cardiac arrest, heart failure, and cardiogenic shock which is defined as persistent hypotension with systolic blood pressure ˂ 90 mmHg for at least 30 minutes, with features of tissue hypoperfusion despite adequate fluid administration [13]. The presence of re-infarction and major bleeding which is defined as ≥ 3g/dl decrease in hemoglobin, transfusion of whole blood or packed RBCs, or an intervention to stop the bleeding within 72 hours post- procedure [14]. No-reflow phenomenon was considered if TIMI flow in the artery ≤ 2, despite successful dilatation and absence of mechanical complications such as dissection, spasm or distal embolization after completing the procedure [15].

## Statistical analysis

Statistical analysis was done using SPSS statistical package (IBM SPSS statistics for windows, version 23, Armnok, NY: IBM Corp.). The normality of the different variables was tested by Shapero Wilks test. The continuous variables were expressed in mean± standard deviation (SD) and medians, while the categorical variables were expressed as frequency and percentages (%). Student’s t test was used in order to test the significance between two groups with normally distributed quantitative variables, while Mann Whitney’s test was used for not normally distributed ones. Chi-square test (χ^2^) was used for categorical variables and whenever any of the expected cells were less than five, Fischer’s exact test was used. Two-sided P. value < 0.05 was considered statistically significant. Univariate binary logistic regression was used to ascertain the effect of possible risk factors on the outcomes of the patients. Multivariate binary logistic regression analysis was performed to identify the independent predictors of CIN development.

## Results

**Figure 1:**
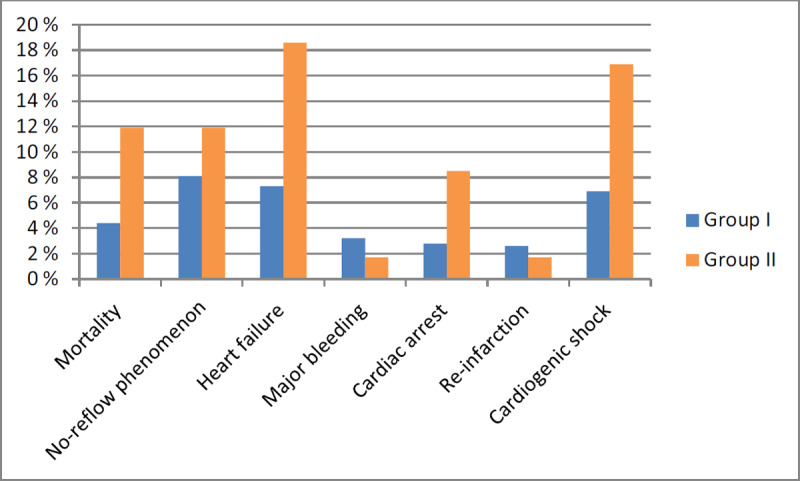
Outcomes of primary percutaneous coronary intervention of both groups.

The present study comprised 550 patients with STEMI who were managed by PPCI in our cardiovascular department. Patients were divided into two groups; group I (Patients without CIN) included 496 patients and group II (Patients with CIN) included 59 patients. The age of patients in group II was significantly higher than in group I. The prevalence of diabetes mellitus was higher in group II than group I with (P. value = 0.001). As regarding history of concomitant diseases and previous medication, there was a statistically significant difference between both groups with more prevalence in group II regarding CKD, atrial fibrillation and NSAIDs use with (P. value = 0.038, 0.041, 0.001 respectively). The volume of contrast agent used during the procedure was significantly higher in group II with (P. value = 0.002). However, there was no statistically significant difference regarding other medication or the culprit vessel between both groups. As regarding mortality and MACE, mortality was higher in group II than group I (11.9% vs. 4.4% respectively, P = 0.015). The incidence of MACE were higher in group II than group I (heart failure; 18.6% vs. 7.3%, cardiac arrest; 8.5% vs. 2.8% and cardiogenic shock; 16.9% vs. 6.9% with P. value = 0.003, 0.024, 0.007 respectively) with no statistically significant difference regarding major bleeding, no-reflow phenomenon and re-infarction between both groups as shown in Table 1 and Figure 1.

**Table 1:** Demographic, clinical characteristics, angiographic results and outcomes of the two groups.

	Group I (No CIN) (N = 496) (89.4%)	Group II (CIN) (N = 59) (10.6%)	P value

Age, years	57.44 ± 10.9	61.42 ± 9.81	0.008*
Male gender, n (%)	296 (59.7%)	30 (50.8%)	0.193
Hypertension, n (%)	172 (34.7%)	21 (35.6%)	0.889
Diabetes mellitus, n (%)	165 (33.3%)	34 (57.6%)	0.001*
Smoking, n (%)	134 (27.0%)	20 (33.9%)	0.264
Dyslipidemia, n (%)	173 (34.9%)	18 (30.5%)	0.504
Prior MI, n (%)	43 (8.7%)	9 (15.3%)	0.101
PVD, n (%)	93 (18.8%)	15 (25.4%)	0.221
CKD, n (%)	61 (12.3%)	13 (22.0%)	0.038*
Height (cm)	167.3 ± 4.76	166.4 ± 5.08	0.181
Weight (kg)	70.85 ± 7.13	70.63 ± 10.0	0.831
BMI (kg/m^2^)	24.54 ± 2.95	25.00 ± 3.57	0.269
Heart rate (bpm)	66.81 ± 10.3	66.54 ± 10.5	0.851
Atrial fibrillation, n (%)	43 (8.7%)	10 (16.9%)	0.041*
Systolic BP, mmHg	123.6 ± 18.2	122.2 ± 30.2	0.590
Diastolic BP, mmHg	81.91 ± 9.02	80.76 ± 18.9	0.427
LVEF, (%)	45.50 ± 4.72	44.31 ± 5.69	0.076
Beta blockers, n (%)	303 (61.1%)	33 (55.9%)	0.444
Antiplatelet, n (%)	350 (70.6%)	39 (66.1%)	0.479
NSAIDs use, n (%)	103 (20.8%)	24 (40.7%)	0.001*
ACEI/ARB, n (%)	260 (52.4%)	34 (57.6%)	0.449
Statins, n (%)	362 (73.0%)	39 (66.1%)	0.264
Volume of contrast agent, (ml)	182.2 ± 68.9	213.2 ± 83.5	0.002*
LM coronary artery, n (%)	7 (1.4%)	1 (1.7%)	0.863
LAD coronary artery, n (%)	210 (42.3%)	21 (35.6%)	0.320
CX coronary artery, n (%)	142 (28.6%)	16 (27.1%)	0.808
RCA coronary artery, n (%)	137 (27.6%)	21 (35.6%)	0.200
Mortality, n (%)	22 (4.4%)	7 (11.9%)	0.015*
No-reflow phenomenon, n (%)	40 (8.1%)	7 (11.9%)	0.322
Heart failure, n (%)	36 (7.3%)	11 (18.6%)	0.003*
Major bleeding, n (%)	16 (3.2%)	1 (1.7%)	0.519
Cardiac arrest, n (%)	14 (2.8%)	5 (8.5%)	0.024*
Re-infarction, n (%)	13 (2.6%)	1 (1.7%)	0.668
Cardiogenic shock, n (%)	34 (6.9%)	10 (16.9%)	0.007*

PVD: peripheral vascular diseases; CKD: chronic kidney diseases; BMI: body mass index; BP: blood pressure; LVEF: left ventricular ejection fraction; NSAIDs: non-steroidal anti-inflammatory drugs; ACEI/ARB: angiotensin converting enzyme inhibitors/angiotensin receptor blockers; LM: left main; CX: circumflex; LAD: left anterior descending; *: significant P value.

**Figure 2:**
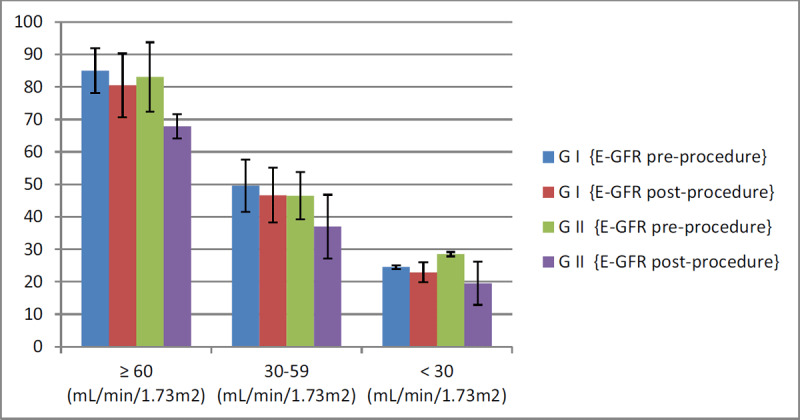
E-GFR pre and post primary percutaneous coronary intervention of both groups.

According to laboratory results, random blood sugar was significantly higher in group II than group I (193.9 ± 83.9 vs. 167.3 ± 71.1 respectively, P. value = 0.008), there was a statistically significant difference between both groups regarding creatinine levels pre-procedure with higher levels in group II than group I (1.154 ± 0.39 vs. 1.062 ± 0.22 respectively, P. value = 0.002) and post-procedure (2.175 ± 0.88 vs. 1.109 ± 0.25 respectively, P. value = 0.001). Similarly, there was a statistically significant difference regarding e-GFR with lower levels in group II than group I with (P. value = 0.001), as shown in Table 2 and Figure 2.

**Table 2:** Laboratory results of the two groups.

	Group I (No CIN) (N = 496) (89.4%)	Group II (CIN) (N = 59) (10.6%)	P value

Hemoglobin level, g/dL	11.46 ± 1.54	11.10 ± 1.69	0.093
Random blood sugar, mg/dl	167.3 ± 71.1	193.9 ± 83.9	0.008*
Creatinine pre-procedure, mg/dl	1.062 ± 0.22	1.154 ± 0.39	0.002*
Creatinine post-procedure, mg/dl	1.109 ± 0.25	2.175 ± 0.88	0.001*
CK-MB, U/L	76.60 ± 37.8	80.20 ± 34.9	0.487
E-GFR pre-procedure, n (%)			
≥60 (mL/min/1.73 m^2^)	377 (76.0%)	32 (54.2%)	0.001*
30–59 (mL/min/1.73 m^2^)	111 (22.4%)	23 (39.0%)	
<30 (mL/min/1.73 m^2^)	8 (1.6%)	4 (6.8%)	
E-GFR post-procedure, n (%)			
≥60 (mL/min/1.73 m^2^)	347 (70.0%)	12 (20.3%)	0.001*
30–59 (mL/min/1.73 m^2^)	129 (26.0%)	33 (55.9%)	
<30 (mL/min/1.73 m^2^)	20 (4.0 %)	14 (23.7%)	
E-GFR pre-procedure, (M ± SD)			
≥60 (mL/min/1.73 m^2^)	84.98 ± 6.92	83.06 ± 10.7	0.001*
30–59 (mL/min/1.73 m^2^)	49.58 ± 8.09	46.46 ± 7.28	
<30 (mL/min/1.73 m^2^)	24.50 ± 0.57	28.50 ± 0.70	
E-GFR post-procedure, (M ± SD)			
≥60 (mL/min/1.73 m^2^)	80.48 ± 9.85	67.83 ± 3.71	0.001*
30–59 (mL/min/1.73 m^2^)	46.67 ± 8.39	36.94 ± 9.83	
<30 (mL/min/1.73 m^2^)	22.90 ± 3.07	19.50 ± 6.63	

CK-MB: Creatine kinase myocardial band; E-GFR: estimated glomerular filtration rate; *: significant P value.

Multivariate regression analysis was performed and identified the following independent predictors of CIN: age > 60 years OR 6.083 (CI95% 3.143–11.77, P = 0.001), presence of diabetes mellitus OR 2.491 (CI95% 1.327–4.675, P = 0.005), non-steroidal anti-inflammatory drugs (NSAIDs) use OR 2.708 (CI95% 1.393–5.263, P = 0.003), volume of contrast agent > 200 ml OR 6.543 (CI95% 3.382–12.65, P = 0.001) and cardiogenic shock OR 4.514 (CI95% 1.738–11.72, P = 0.002) as shown in Table 3.

**Table 3:** Multivariate regression analysis showing the independent predictors of contrast induced nephropathy.

	Multivariate analysis		P value

	OR	(95% CI)	

Age > 60 years	6.083	3.143–11.77	0.001*
Diabetes mellitus	2.491	1.327–4.675	0.005*
Atrial fibrillation	1.087	0.259–4.561	0.910
Chronic kidney diseases	1.934	0.527–7.100	0.320
NSAIDs use	2.708	1.393–5.263	0.003*
Volume of contrast agent > 200 ml	6.543	3.382–12.65	0.001*
Cardiogenic shock	4.514	1.738–11.72	0.002*
Heart failure	2.216	0.920–5.341	0.076
Cardiac arrest	2.688	0.716–10.09	0.143

OR: Odds ratio; CI: confidence interval; NSAIDs: non-steroidal anti-inflammatory drugs; *: significant P value.

## Discussion

Primary PCI is the preferred method of reperfusion in patients with STEMI. The present study was conducted on 550 patients with STEMI managed by PPCI in our cardiovascular department. The incidence of CIN varies greatly following PPCI depending on the baseline demographic and clinical characteristics of the patients, as well as the circumstances of the angiographic procedure. Our study showed the incidence of CIN was 10.6% of the whole patients subjected to PPCI. This result was nearly similar to the results of other previous investigators e.g., Mehran et al. [16], showed that the overall occurrence of CIN in 8.357 patients was 13.1%. Bouzas-Mosquera et al. [17], reported that the incidence of CIN was 12% in a high risk group of patients undergoing PPCI. Nash et al. [18], stated that radiographic contrast media were responsible for 11% of cases of hospital-acquired renal insufficiency.

Patients with pre-existent CKD, that is defined as an estimated glomerular filtration rate (e-GFR) < 60 mL/min/1.73 m^2^ for greater than three months, is the most commonly recognized risk factor for CIN [5]. In this study patients with CKD were more predominant in the CIN group and represent 22% of all patients in this group. Moreover, patients with atrial fibrillation were higher in the CIN group. Atrial fibrillation is one of the commonest arrhythmias occurring in the setting of STEMI with incidence varying from 5% to 18%. The occurrence of atrial fibrillation in such patients is associated with increased morbidity and mortality as atrial fibrillation can lead to a fall in cardiac output. Potential consequences include a fall in blood pressure and pulmonary congestion, all of which are manifestations of heart failure. This hemodynamic compromise can decrease renal perfusion with more liability to CIN development [19,20,21].

The use of NSAIDs is not obviously associated with an increased risk of CIN in the medical literature. Since these drugs are intrinsically nephrotoxic, there could be a synergistic action with contrast agents for CIN development. The duration and dose of NSAID use should be considered when assessing the patient’s risk. In the present study, we found that patients with NSAID use were significantly higher in the CIN group and multivariate regression analysis showed NSAID use as an independent predictor of CIN. In disagreement with our results, Diogo et al., stated that there is no association between NSAID use and CIN development [22]. Other independent predictors of CIN in multivariate regression analysis in our study were age > 60 years. Advanced age is often associated with a decline in e-GFR, with more comorbidity in old age and the severity of coronary artery disease is higher with a longer procedural time. Diabetes mellitus with associated hyperglycemia can lead to microvascular renal lesions and diabetic nephropathy. Cardiogenic shock is responsible for volume depletion and decreased renal perfusion [23]. In agreement with our results, Newhouse et al. [24], reported that CIN largely depends on coexisting risk factors such as intravascular volume depletion, diabetes mellitus, baseline renal dysfunction and heart failure.

Contrast media isn’t the sole factor responsible for renal impairment in patients undergoing PPCI. Multiple contributing factors may lead to renal dysfunction or potentiate the effects of contrast media e.g. hypotension, hemodynamic instability and acute heart failure [16]. However, higher doses of contrast media are a risk factor for the occurrence of CIN, and our results confirmed this hypothesis. The volume of contrast media used during the procedure was significantly higher in group II (213.2 ± 83.5) versus (182.2 ± 68.9) in group I with (P. value = 0.002). In agreement with our results Kooiman et al., stated that a high dose of contrast was a marginal predictor of CIN after PPCI [25]. Several studies also reported a positive correlation between higher doses of contrast media and the incidence of CIN in patients undergoing PPCI [26,27].

## Conclusion

Contrast induced nephropathy is a common complication in patients with STEMI undergoing PPCI, even with normal baseline renal function. The independent predictors of CIN were advanced age, presence of diabetes mellitus, NSAIDs use, the volume of contrast agent >200 ml and cardiogenic shock. Contrast induced nephropathy was associated with higher morbidity and a very high mortality rate. Thus, newer preventive strategies of renal protection during PPCI are warranted, particularly in high risk patients.
